# Comparison of different endoscopic resection techniques for submucosal tumors originating from muscularis propria at the esophagogastric junction

**DOI:** 10.1186/s12876-019-1099-5

**Published:** 2019-11-06

**Authors:** Hong-wei Xu, Qi Zhao, Shu-xia Yu, Ying Jiang, Jing-hua Hao, Bin Li

**Affiliations:** Department of Gastroenterology, Shandong Provincial Hospital affiliated to Shandong University, No. 324, Jingwuweiqi Road, Jinan, Shandong China

**Keywords:** Esophagogastric junction, Submucosal tumor, Muscularis propria, Submucosal tunneling endoscopic resection, Endoscopic submucosal excavation

## Abstract

**Background:**

To compare the outcomes of submucosal tunneling endoscopic resection (STER) and submucosal excavation (ESE) for the treatment of submucosal tumors (SMTs) arising from the muscularis propria (MP) at the esophagogastric junction (EGJ).

**Methods:**

A retrospective analysis of patients with SMTs at EGJ who underwent STER and ESE from October 2011 to October 2017 was performed. The outcomes evaluated were operation time, complete resection rate, adverse events, and tumor recurrence.

**Results:**

Ninety patients were included in this study. Complete resection rates in the STER group were higher than those of the ESE group (100 vs. 92%, *p* < 0.05). For tumors ≤15 mm, both techniques achieved 100% complete resection rate; but for tumors > 15 mm, complete resection rate was higher in the STER group than the ESE group (100% vs. 77.8%, *p* < 0.05). Subgroup analyses revealed that the operation time of STER for in cardiac-gastric group was longer than that for ESE (145.14 ± 42.43 min vs. 70.32 ± 39.84 min*, p* <  0.05). The air leakage symptoms were more frequent in STER group (90.9% vs. 50.0%, *p* < 0.05). No tumor recurrence occurred in both the STER and ESE groups.

**Conclusions:**

For SMTs ≤15 mm, both STER and ESE have similar satisfactory therapeutic outcomes. However, in the cardiac-gastric subgroup, STER had a longer operative time compared to the ESE procedure. For SMTs > 15 mm, STER is the preferred choice due to its higher complete resection rate.

## Background

Submucosal tumors (SMTs) at the esophagogastric junction (EGJ) are defined as the submucosal tumors located partially or fully within 1 cm proximal and 2 cm distal to the esophagogastric junction (squamo-columnar junction) [1]. SMTs are usually covered with normal gastrointestinal mucosa, and most patients have no specific clinical manifestations [2]. Moreover, some mesenchymal neoplasms, including gastrointestinal stromal tumors (GISTs), have malignant potential [3, 4]. Thus, surgical resection is often suggested, especially for high-risk tumors or patients who are greatly worried about their long-term prognosis.

Previously, surgical wedge resection was the preferred option for SMTs [5]. With advancements in technologies and techniques, therapeutic endoscopic procedures have evolved as alternative approaches for the excision of SMTs [6–9]. It has been reported that gastrointestinal SMTs, especially those originating from the muscularis mucosa ventriculi and submucosa, can be successfully removed by endoscopic submucosal dissection (ESD), endoscopic submucosal excavation (ESE), and endoscopic full-thickness resection (EFR) [10]. The ESE technique involves making a tiny incision of the mucous membrane above the lesion and dissection the tumor directly. Nevertheless, for SMTs arising from muscularis propria (MP) layer, particularly those growing outside the cavity and densely adhered to the serosal layer, complete resection is difficult and risky [11]. Currently, submucosal tunneling endoscopic resection (STER) is well developed for the treatment of these SMTs [12]. Inspired by these good outcomes, we began using STER for endoscopic resection of SMTs arising from the MP layer of the EGJ. The advantage of this tunneling technique is that it maintains the integrity of the surrounding mucosa of the lesion. With the tunneling technique, the lesion is not required to be in a horizontal plane with the mucous cut which may maintain the integrity of the surrounding mucosa. The direct ESE is also a good option for resection of the tumor due to its shorter operative time. Till now, very few studies on the excision of SMTs originating from the MP layer of the EGJ have been reported [13]. The purpose of this study was to compare the efficacy and safety of STER and ESE for resection of SMTs.

## Methods

### Patients

This was a retrospective study involving consecutive patients admitted to Department of Gastroenterology, Shandong Provincial Hospital affiliated to Shandong University between October 2011 and October 2017. This study was approved by the Institutional Review Board of Shandong Provincial Hospital affiliated to Shandong University. Written informed consent was obtained from each participant before surgery. Patients who met the following criteria were included: (1) SMTs located partially or fully at the EGJ with origin from the MP layer as confirmed by endoscopic ultrasonography (EUS); (2) the maximum diameter of the tumor less than 3.0 cm. The exclusion criteria were as follows: (1) lesions originating from the mucosal/submucosal layer; (2) patients unfit for general anesthesia; (3) predominant extraluminal growth was shown by EUS or computed tomography (CT).

### Grouping

**Fig. 1:**
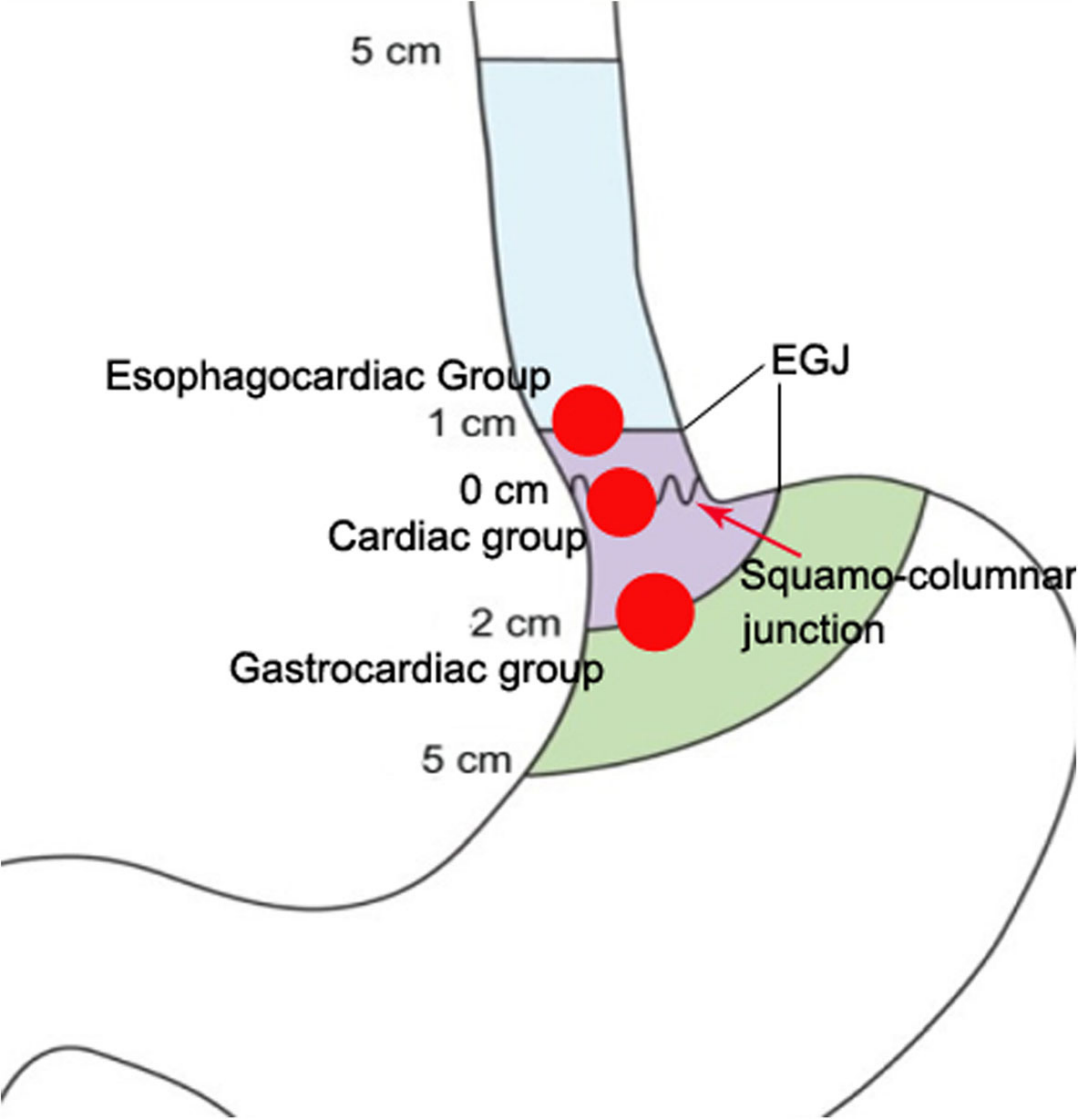
Grouping of the patients with submucosal tumors. The patients were classified into the cardiac group if the tumor’s center was within the anatomic EGJ and straddled the squamo-columnar junction. The patients were classified into the esophagocardiac group if the tumor was partially located above the anatomic EGJ and its distal edge failed to reach the squamo-columnar junction. The patients were classified into the gastrocardiac group if the tumor was partially located below the anatomic EGJ and its proximal edge failed to reach the squamo-columnar junction. EGJ, esophagogastric junction, was defined as the area within 1 cm above and 2 cm below the squamo-columnar junction

The patients were classified into three groups based on the location of the lesions: esophagocardiac, cardiac, and cardiac-gastric groups (Fig. 1). In the esophagocardiac group, the tumor was partially located within the EGJ area and its distal margin failed to reach the squamo-columnar junction. The tumor located within the anatomic EGJ and straddled the squamo-columnar junction were considered as cardiac group. The tumor partially locating below the anatomic EGJ and its proximal margin failed to reach the squamo-columnar junction were included into cardiac-gastric group.

### ESE procedure

ESE was performed under general anesthesia. Antibiotics were given intravenously 0.5 h before the procedure. The operation was conducted using a single and/or dual-channel endoscope (Olympus).

**Fig. 2:**
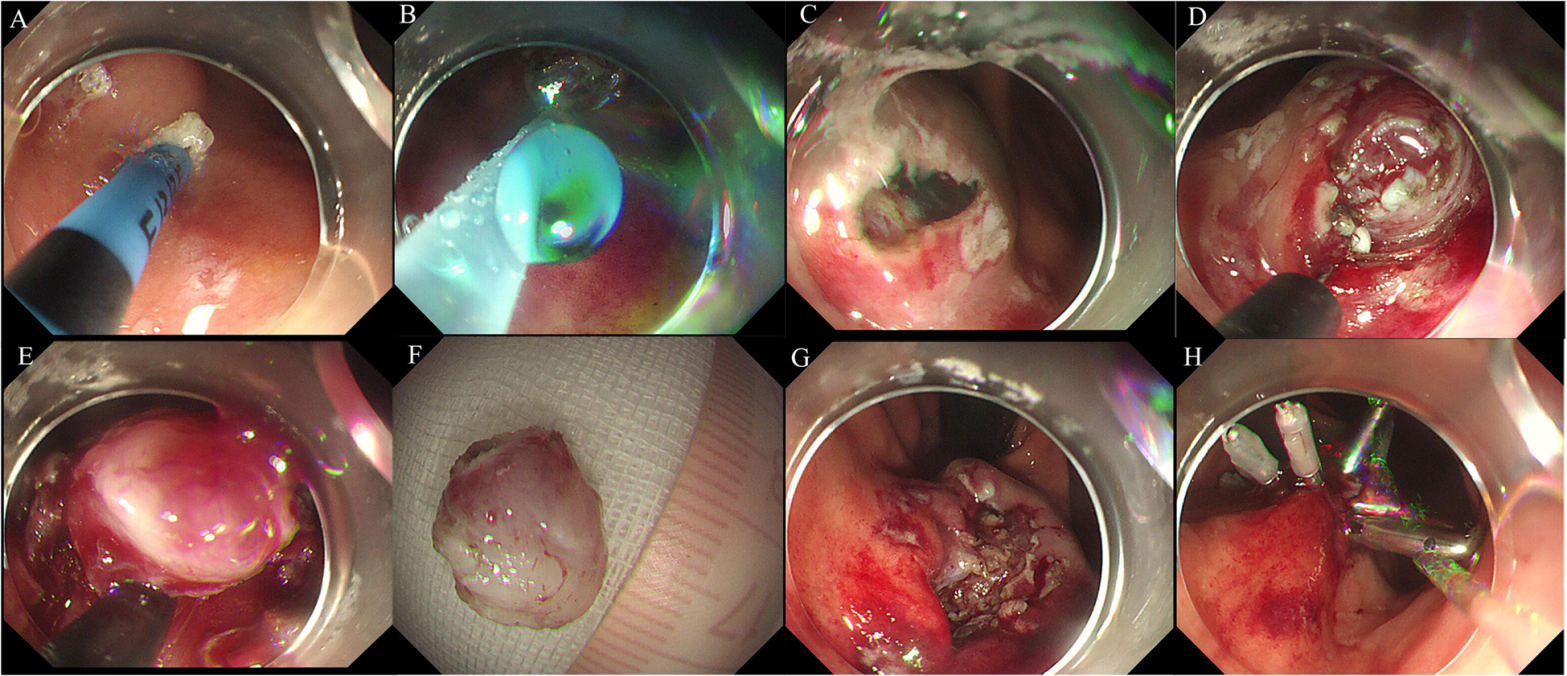
Procedure of endoscopic submucosal excavation. **a**: Marking of the submucosal tumor with argon knife; **b**: Submucosal injection with fluid mixture; **c**: Incision of the covering mucosa; **d**: Identification of the submucosal tumor; **e**, **f**: Peeling off the lesions; **g**: The wound after resection; **h**: Closure of the mucosal incision by clips

The operative steps were as follows:

1. Marking of the tumor location (Fig. 2a): Argon or coagulation was used to mark the oral and anal end of the target lesion.
2. Submucosal injection (Fig. 2b ,c): A solution prepared using saline, indigo carmine and epinephrine was injected into submucosa to elevate the lesion so as to facilitate separation of the covering mucosal and submucosal layer from the lesion.
3. Exposure of the lesion (Fig. 2d): The covering mucosa was incised longitudinally using a dual or hook knife over the tumor along the marked site, followed by separation of the submucosa to expose the tumor using endoscopic submucosal dissection.
4. Peeling of the lesion (Fig. 2e, f): After dissecting the submucosal and muscular tissue around the tumor capsule, the lesion was peeled with IT_2_ or Hook knife.
5. Wound treatment (Fig. 2g, h): Hot biopsy forceps was used for achieving hemostasis during the operation. The small blood vessels were burned by argon. If the residual cavity was deep, the mucosal incision was approximated by clips to avoid delayed hemorrhage or perforation.

### STER procedure

**Fig. 3:**
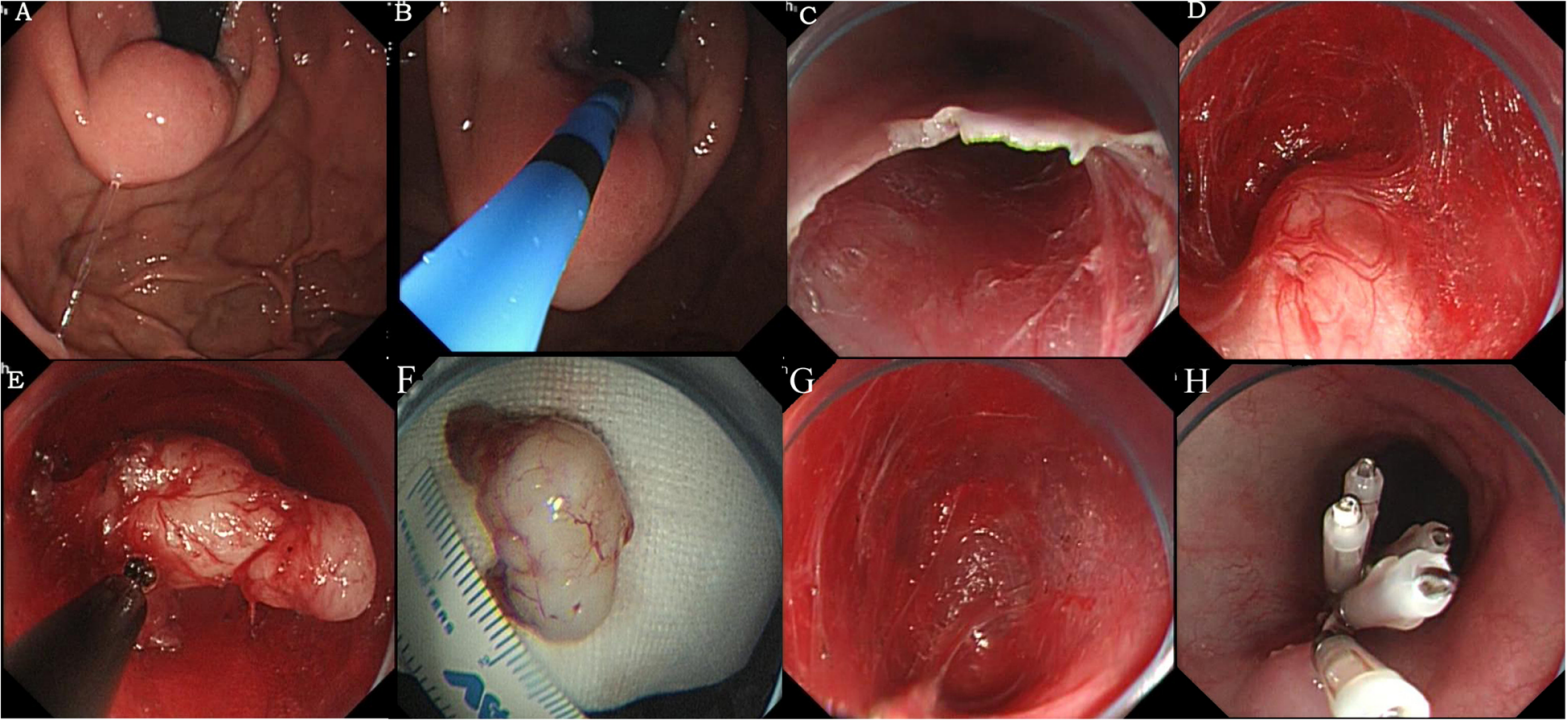
Procedure of submucosal tunneling endoscopic resection. **a**: The submucosal tumor; **b**: Marking of the tumor with argon knife; **c**: Creation of the submucosal tunnel; **d**: Visualization of the submucosal tumor; **e**: Dissection of the tumor from the muscular layer within the submucosal tunnel; **f**: The lesion; **g**: The wound after resection; **h**: Closure of the mucosal incision with clips

The complete procedure of STER has been described previously by Xu et al. [6] In this study, the procedure was modified as below:

1. Localization of the tumor (Fig. 3a): Argon or dual knife was used to mark the tumor (Fig. 3b).
2. Construction of a submucosal tunnel (Fig. 3c): After injecting a fluid cushion about 5 cm proximal to the SMT, a 2-cm longitudinal mucosal entry point was made by using a dual or hook knife on the esophageal mucosa. A submucosal longitudinal tunnel was then created with dual knife. The tunnel terminated at about 1–2 cm distal to the tumor to provide good endoscopic view of the SMT and sufficient space for dissection.
3. Exposure of the tumor (Fig. 3d): The level of difficulty of exposing the SMT depends on its location. Local injection of methylene blue in the original SMT before endoscopic tunneling was performed to facilitate identification of the tumor.
4. Resect the SMT. Separation of the MP layer was performed by using an IT_2_ or Hook knife (Fig. 3e, f). complete resection of the tumor including its capsule was performed. Damage to the esophageal adventitia or gastric serosa was avoided.
5. Hemostasis (Fig. 3g): After tumor resection, hot biopsy forceps were used to control hemorrhage and exposed small arteries in order to avoid late onset bleeding [14].
6. Closure of the mucosal incision (Fig. 3h).

### Postoperative management and follow up

Postoperative management included monitoring for chest pain, dyspnea, and other discomforts [13–15]. Possible complications included gas leakage into the surrounding tissue or lacuna, hemorrhage, perforation, infection, and pain. Once perforation, subcutaneous emphysema, or pneumothorax occurred, antibiotics were administered for as long as 48 h after surgery. The patient’s body temperature was closely monitored, and blood culture testing was performed. When patients complained of severe pain, they were monitored for signs of infection or perforation. The main outcomes included the complete resection rate, the tumor recurrence rate, and the rate of complications including subcutaneous emphysema, pneumothorax, pneumoperitoneum. Follow-up esophagogastroscopy and EUS were performed at an interval of 3 months during the first year after surgery, and every 6 months during the following 2–5 years to evaluate wound healing and timely identification of any residual or recurrent tumor. The CT scan was used in cases of GIST to detect distant metastasis.

### Statistical analysis

SPSS 16.0 software (SPSS, Chicago, IL, USA) was used for all statistical analyses. The Student t-test, Chi square test, and Fisher’s exact test were used for the comparisons between the groups. Data were presented as mean ± standard deviation. A difference was considered significant when *p* <  0.05.

## Results

### Clinical characteristics

There were 90 patients having 94 SMTs included in this study. The details of the patients and SMTs in both the STER and ESE groups are listed in Table 1. There was no significant difference in the age between the two groups. The male:female ratio was higher in STER group than in ESE group. In the STER group, one patient had three SMTs and two patients had two SMTs. The mean resected lesion size was 22.05 ± 7.67 mm. For six patients, the size of the lesions was < 1 cm (For details, see the Additional file 1: Tables S1 and S2). But due to the fear of malignancy, these six patients had severe anxiety and insisted for endoscopic resection. The main pathology type was leiomyoma (70.45%).

**Table 1:** Detailed information about the patients and SMTs of STER and ESE group

Detailed information	STER	ESE	*P* value
Characteristics of patients			
Number	40	50	/
Average age (years)	52.05 ± 10.15	53.06 ± 8.84	0.143, NS ^a^
Gender ratio (M/F)	22/18	16/34	0.028
Characteristics of tumor			
Number	44	50	/
Size (mm)	22.05 ± 7.667	15.32 ± 7.770	0.000
Location (%)			
Esophagus cardiac	14 (31.82)	6 (12)	/
Cardiac	14 (31.82)	6 (12)	/
Cardiac-gastric fundus	16 (36.36)	38 (76)	0.000
Pathological type (%)			
Leiomyoma	31 (70.45)	34 (68.00)	/
GIST	10 (22.73)	14 (28)	/
Schwannoma	2 (4.55)	2 (4)	/
Lipoma	1 (2.27)	0	0.699, NS ^a^

*M/F* Male/Female, *GIST* gastrointestinal stromal tumor, *ESE* endoscopic submucosal excavation, *STER* submucosal tunneling endoscopic resection; ^a^No Significance

In ESE group, the mean resected lesion size was 15.32 ± 7.77 mm. The main pathology type was leiomyoma (68.00%). Histological examination showed that the majority of the lesions below the cardia to be GISTs, whereas those above the squamo-columnar junction were leiomyomas.

### Clinical outcomes

The characteristics and treatment outcomes of STER and ESE are summarized in Table 2 respectively. The overall complete resection rate in STER was significantly higher than in ESE (ESE: 92%, STER 100%, *p* <  0.05). In four cases, the lesions were too large and deep to perform en bloc resection. In these cases, a part of the lesion was resected for pathological examination which confirmed them to be GIST. Two of the four cases had delayed bleeding and underwent surgical excision. The remaining two patients refused further treatment. The mean time required to resect SMTs was shorter with ESE compared to STER (69.40 ± 39.68 min vs. 104.90 ± 49.59 min*, p* <  0.05).

**Table 2:** Comparison of clinical outcomes between STER and ESE

Clinical outcomes	STER	ESE	*P* value
Number of lesions	44	50	
Complete resection (%)	100	92	< 0.05
Mean procedure time, min (range)	104.9000 ± 49.59105 (37.00–185.00)	69.4000 ± 39.68344 (29.00–160.00)	< 0.05
Histology diagnosis (%)			
Leiomyoma	31 (70.45)	34 (68.00)	
Spindle cell type tumors (Immunohistochemical staining showed leiomyoma)	3 (6.82)	0	
GIST	10 (22.72)	14 (28.00)	
Very low malignant	4 (9.09)	0	
Low malignant	6 (13.64)	8 (16.00)	
Intermediate malignant	0	6 (12.00)	
Schwannoma	2 (4.54)	2 (4.00)	
Lipoma	1 (2.27)	0	
Complications (%)	22	8	< 0.05
Subcutaneous emphysema	12 (54.55)	2 (25.00)	
Mediastinal emphysema	0	0	
Pneumothorax	6 (27.27)	2 (25.00)	
Pneumoperitoneum	2 (9.09)	0	
Hemorrhage	0	2 (25.00)	
Perforation	0	0	
Nasal bleeding	2 (9.09)	0	
Delayed bleeding	0	0	
Fever	0	2 (25.00)	
Median follow-up period, month (range)	17.5250 ± 14.3169 (6.00–48.00)	11.9600 ± 6.5371 (3.00–24.00)	
Tumor recurrence	0	0	NS^a^

*GIST* gastrointestinal stromal tumor, *ESE* endoscopic submucosal excavation, *STER* submucosal tunneling endoscopic resection; ^a^No Significance

Mediastinal emphysema/infection was not observed in either of the groups. In ESE group, pneumothorax occurred in two patients. These cases were treated by closed thoracic drainage for three days. In two cases, delayed hemorrhage occurred one day after complete resection. The bleeding was controlled by endoscopic spraying of Monsel’s Solution (Monsel’s salt, a ferrous sulfite solution) over the bleeding point. Two cases developed fever two days after the operation which was managed by three days of antibiotics therapy (Table 2). In the STER group, subcutaneous emphysema and pneumothorax developed in 12 and 6 patients respectively. The incidence of subcutaneous emphysema with STER was significantly higher than ESE (Table 2).

### Subgroup analysis based on the tumor location

In the STER group, the complication rate in the esophago-cardiac group was remarkably lower than that in the cardiac and the cardiac-gastric groups. The mean operation time in the esophago-cardiac and cardiac groups was significantly shorter in contrast with that of the cardiac-gastric group (Table 3).

**Table 3:** Subgroup analysis based on the tumor location of STER and ESE group

	STER group	ESE group	*P* value
Complications rate (%)			
Esophagus-cardia	0/14 (0)	2/6 (33.33)	
Cardia	6/12 (50)	0/6 (0)	
Cardiac-gastric fundus	8/14 (57.14)	4/38 (10.53)	
P^1^	0.003	0.121, NS^a^	
P^2^	0.001	0.130, NS^a^	
P^3^	0.716, NS^a^	0.405, NS^a^	
Operating time (min)			
Esophagus-cardia	83.43 ± 23.14408	83.00 ± 54.75	NS^a^
Cardia	83.00 ± 52.79463	50.00 ± 2.68	NS^a^
Cardiac-gastric fundus	145.1429 ± 42.43159	70.3158 ± 39.84	< 0.05
P^1^	0.980, NS^a^	0.200, NS^a^	
P^2^	0.000	0.49, NS^a^	
P^3^	0.003	0.004	

*STER* submucosal tunneling endoscopic resection, *ESE* endoscopic submucosal excavation; ^a^No Significance; P^1^, the comparison of esophagus-cardia and cardia; P^2^, the comparison of esophagus-cardia and cardia-gastric; P^3^, the comparison of cardia-gastric and cardia

In the ESE group, the complication rate in the esophago-cardiac group was higher than that in the cardiac and the cardiac-gastric groups. The operation time in the cardiac-gastric group was significantly longer than the cardiac group (*p* = 0.004, Table 3). As shown in the Table, the mean operating time of STER and ESE were similar in the esophago-cardiac and cardiac groups. But in the cardiac-gastric group, ESE had a shorter mean operating time.

### Subgroup analysis based on tumor size

We divided the patients with tumor size > 15 mm and ≤ 15 mm into separate groups (Table 4). For tumors ≤15 mm, the complete resection rate with both ESE and STER was 100% without perforation. The mean operating time was longer with STER than ESE. For tumors > 15 mm, STER group had a higher complete resection rate but also a higher complication rate than ESE group. Both groups had similar operating time.

**Table 4:** Subgroup analysis on clinical outcomes of ESE and STER

	STER	ESE	*P* value
Lesion size ≤15 mm			
Number	13	32	
Mean tumor size (mm)	11.85 ± 2.41	10.19 ± 2.25	0.033
Operation time (min)	102.85 ± 55.99	55.63 ± 35.22	0.032
Complete resection rate (%)	13/13 (100)	32/32 (100)	1.000, NS^a^
Perforation rate (%)	0	0	
Complications rate (%**)**	10/11 (90.9) ^b^	4/32 (12.5)	< 0.05
Subcutaneous emphysema	6	0	
Pneumoperitoneum	2	0	
Pneumothorax	2	0	
Bleeding	0	2	
Fever	0	2	
Lesion size > 15 mm			
Number	31	18	
Mean tumor size (mm)	26.32 ± 4.24	24.44 ± 5.204	0.18, NS^a^
Operation time (min)	112.58 ± 47.75	93.89 ± 35.85	0.21, NS^a^
Complete resection rate (%)	31/31 (100)	14/18 (77.8)	< 0.05
Perforation rate (%)	0	0	
Complications rate (%)	12/29 (41.4)^c^	4/18 (22.2)	< 0.05
Subcutaneous emphysema	6	2	
Pneumoperitoneum	0	0	
Pneumothorax	4	2	
Bleeding	2	0	
Fever	0	0	

*ESE* endoscopic submucosal excavation, *STER* submucosal tunneling endoscopic resection; ^a^No Significance; ^b^In one case, there were three lesions (≤ 15 mm), therefore the case number with lesion size ≤15 mm was 11, while the lesion number was 13; ^c^ In two cases, each had two lesions (> 15 mm), therefore the case number with lesion size > 15 mm was 29

### Follow up outcomes

Mucosal cicatrization without tumor recurrence were visible in all patients who underwent complete excision on follow-up endoscopy at 3, 6, 12, and 24 months after the procedure of STER or ESE.

## Discussion

Gastrointestinal SMTs are mostly leiomyomas and GISTs which can be differentiated based only on histopathological examination [16]. Currently, the best diagnostic method is endoscopic ultrasound-guided fine-needle aspiration [17]. The treatment option is controversial if the SMT is suspected to be a GIST. According to the 2017 National Comprehensive Cancer Network guidelines, surgical resection is recommended for the GISTs with symptoms, those > 2 cm in diameter, or < 2 cm but with suspicious EUS features such as irregular border, cystic areas, ulceration, echogenic foci, and heterogeneity. While the 2012 European Society for Medical Oncology guidelines recommend all histologically confirmed GIST to be resected [16], long-term follow-up is suggested as even low-risk GIST can recur as long as 10 years later if you do not choose surgical resection [18].

As a minimally invasive approach, endoscopic resection is a good alternative for upper gastrointestinal SMTs with the advantages being mild surgical trauma, rapid recovery, and fewer adverse effects on digestive tract function. Additionally, endoscopic resection can remove the tumor completely and provide specimens for pathological examination [19, 20]. Endoscopic resection includes ESE, EFR, and STER. STER, first proposed by Chinese scholars, is based on the principles of endoscopic tunneling technique mainly useful in resecting esophageal and gastric cardiac SMTs and is an innovative application of ESD. Compared with other endoscopic approaches, STER has some prominent advantages. First, the integrity of the mucosa and submucosa can be restored by closing the mucosal incision with several clips. Second, the 5-cm-long submucosal tunnel acts as a safeguard against post-procedure leak and prevents the development of digestive tract fistulae or pleural/abdominal infection. Third, due to good visualization of the MP layer in the submucosal tunnel, precise hemostasis can be easily achieved [6]. However, the tunneling technique itself has its drawbacks, since relatively straight and relatively fixed gastrointestinal lumen are required for the construction of tunnel, and not all parts of the digestive tract are suitable for the submucosal tunnel creation. In addition, STER requires advanced training and a long operating time while ESE is a technical extension of ESD, a commonly used procedure to excise early-stage carcinoma. For lesions involving the entire thickness of the muscle, EFR is one of the endoscopic therapeutic options. For small lesions, after EFR the size of defect is small and can be easily closed by endoscopy. But for larger lesions, it is difficult to seal the perforation by endoscopy especially those wound close to the gastroesophageal junction and the cardia. Hence, we seldom use EFR method for larger lesions located near the gastroesophageal junction. In such cases we use ESE or STER. The advantage of ESE is that ESE can be used for most of the gastrointestinal SMTs and the operation time is relatively short compared to STER. Furthermore, based on the available literature, STER does not have considerable advantages over ESE either in the complete resection rate or in the incidence of adverse events [19–25]. Due to stenosis, sharp corners, special anatomical structure, small endoscopic space and technical difficulties, SMTs arising from muscularis propria are difficult to treat with endoscopy. Endoscopic resection technique based on ESD (ESE, STER) is safe and effective for the treatment of SMTs which arise from EGJ muscularis propria [26–29]. However, few studies have focused on the application and the comparison between endoscopic therapies such as STER and ESE in SMTs originating from the MP layers at the EGJ. Also, there is no comparative study focusing on the outcomes of different subtypes of SMTs at EGJ.

Our team developed ESE, STER technology based on the ESD. With SMTs at EGJ, the complete resection rate was higher in STER than that in ESE for the large tumor (≥ 1.5 cm) due to physiological cardiac stenosis. In addition, the mean operating time in STER group was longer than that in ESE. Especially in cardiac-gastric subgroup, STER operating time was significantly longer than the ESE. Because of the special location, for the SMTs located near the greater curvature of the cardia, we needed more time to create the submucosal tunnel as the tunnel mucosa was liable to be damaged and it is more difficult to find the lesion. However, we created the submucosal tunnel safely in most cases by careful performance. The incidence of postoperative complications was higher in STER. The main complications were the subcutaneous emphysema, pneumothorax and pneumoperitoneum due to leakage of the gas into interstitial space during the procedure. But all these complications could be easily managed because of the use of carbon dioxide gas. For the tumor closely related with the serosa, in order to ensure the complete removal of the tumor, the entry of the gas into the interstitial space was inevitable. In all complete resection cases, no postoperative gastrointestinal fistula occurred.

As the tumor size increases, the endoscopic resection time, the difficulty of the operation, risk of perforation, and delayed bleeding risk also increases [30]. For tumors less than 15 mm in diameter, both ESE and STER had high complete resection rate. Operation time was significantly longer in STER, especially in cardiac-gastric group.

There are some limitations of our research. This was a retrospective study in which selection bias is inevitable. Our initial experience with STER included a limited sample size, which constituted another weakness. Additionally, the 2-year follow-up was not enough to determine long-term results.

## Conclusions

In conclusion, for SMTs ≤15 mm, both ESE and STER had similar efficacy, but the operative time was shorter for ESE. For SMTs > 15 mm, particularly irregularly shaped lesions, crossing the cardia, ESE may not resect the SMT completely and may be technically difficult to close the mucosal incision. Hence, STER is recommended as the preferred method of treatment due to its higher complete resection rate, although the operation time for STER is longer and finding the lesion in the fundus of the stomach though the submucosal tunnel is more difficult.

## Supplementary information

**Additional file 1: Table S1.** Clinicopathological characteristics and treatment outcomes of submucosal tumors of the esophagogastric junction originating from the muscularis propria layer treated by STER. **Table S2.** Clinicopathological characteristics and treatment outcomes of submucosal tumors of the esophagogastric junction originating from the muscularis propria layer treated by ESE.

## Data Availability

The datasets generated and analyzed during the present study are available from the corresponding author on reasonable request.

## Abbreviations

EFR: Endoscopic full-thickness resection
EGJ: Esophagogastric junction
EMR: Endoscopic mucosal resection
ESD: Endoscopic submucosal dissection
ESE: Endoscopic submucosal excavation
ESMR: Endoscopic submucosal resection
EUS: Endoscopic ultrasonography
GIST: Gastrointestinal Stromal Tumors
MP: Muscularis propria
SMTs: Submucosal tumors
STER: Submucosal tunneling endoscopic resection
