# Supplementary material for: Comparison of different endoscopic resection techniques for submucosal tumors originating from muscularis propria at the esophagogastric junction
Source: BMC Gastroenterol. 2019 Nov 6;19:174. doi: 10.1186/s12876-019-1099-5 (PMC6833169; doi:10.1186/s12876-019-1099-5)
Supplement: Supplementary file 1 — Additional file 1: Table S1. Clinicopathological characteristics and treatment outcomes of submucosal tumors of the esophagogastric junction originating from the muscularis propria layer treated by STER. Table S2. Clinicopathological characteristics and treatment outcomes of submucosal tumors of the esophagogastric junction originating from the muscularis propria layer treated by ESE. [file 12876_2019_1099_MOESM1_ESM.docx]

**Additional file 1: Table S1** Clinicopathological characteristics and treatment outcomes of submucosal tumors of the esophagogastric junction originating from the muscularis propria layer treated by STER

| **Case.**  **sex/**  **Age.y** | **Tumor**  **location** | **Tumor size(mm)**  **average** | **En bloc resection** | **Operating time (min)**  **average** | **Full MP defect^a^** | **Complication** | **Pathologic diagnosis** | **Follow-up time**  **(mouth)** |
| --- | --- | --- | --- | --- | --- | --- | --- | --- |
| 1.M/32 | Cardia | 15/11/11（three SMTs） | Yes | 145 | NO | SE | Leiomyoma | 48 |
| 2.F/54 | Cardia | 20 | Yes | 60 | NO | NO | Leiomyoma | 48 |
| 3.M/40 | Cardia -Gastric fundus | 20 | Yes | 66 | Yes | SE/ Pt | GIST( Very low malignant ) | 48 |
| 4.F/65 | Cardia | 15 | Yes | 162 | Yes | SE/ Pp | Spindle cell type tumours ( Leiomyoma) | 48 |
| 5.M/69 | Esophago-Cardia | 10 | Yes | 54 | NO | NO | Leiomyoma | 18 |
| 6.F/55 | Esophago-Cardia | 30 | Yes | 71 | NO | NO | Leiomyoma | 18 |
| 7.F/58 | Cardia -Gastric fundus | 30/25（two SMTs） | Yes | 180 | Yes | SE/ Pt | Leiomyoma/  Lipoma | 18 |
| 8.M/49 | Esophago-Cardia | 30 | Yes | 110 | NO | NO | Leiomyoma | 14 |
| 9.F/49 | Cardia | 25 | Yes | 49 | NO | NO | Leiomyoma | 14 |
| 10.F/53 | Cardia | 10 | Yes | 37 | NO | SE/ Pt | Leiomyoma | 12 |
| 11.M/65 | Esophago-Cardia | 20 | Yes | 83 | Yes | NO | Schwannoma | 12 |
| 12.M/34 | Esophago-Cardia | 30 | Yes | 120 | NO | NO | Leiomyoma | 10 |
| 13.F/57 | Cardia | 12 | Yes | 45 | NO | NO | Leiomyoma | 9 |
| 14.M/59 | Cardia-Gastric lesser curvature | 20 | Yes | 115 | NO | NO | GIST( Very low malignant ) | 9 |
| 15.M/34 | Cardia -Gastric fundus | 30 | Yes | 185 | NO | NO | GIST(low malignant) | 9 |
| 16.M/38 | Cardia -Gastric fundus | 20 | Yes | 66 | Yes | SE/ Pt | GIST( Very low malignant ) | 32 |
| 17.F/56 | Cardia | 20 | Yes | 60 | NO | NO | Leiomyoma | 48 |
| 18.M/34 | Cardia | 15 | Yes | 145 | NO | SE | Leiomyoma | 48 |
| 19.F/61 | Cardia | 15 | Yes | 162 | Yes | SE/ Pp | Spindle cell type tumours ( Leiomyoma) | 32 |
| 20.F/56 | Cardia | 10 | Yes | 37 | NO | SE/ Pt | Leiomyoma | 12 |
| 21.M/62 | Esophago-Cardia | 20 | Yes | 83 | Yes | NO | Schwannoma | 12 |
| 22.F/51 | Esophago-Cardia | 30 | Yes | 71 | NO | NO | Leiomyoma | 18 |
| 23.M/69 | Esophago-Cardia | 10 | Yes | 54 | NO | NO | Leiomyoma | 18 |
| 24.F/58 | Cardia | 12 | Yes | 45 | NO | NO | Leiomyoma | 10 |
| 25.M/51 | Esophago-Cardia | 30 | Yes | 110 | NO | NO | Leiomyoma | 14 |
| 26.F/47 | Cardia | 25 | Yes | 49 | NO | NO | Leiomyoma | 14 |
| 27.M/33 | Esophago-Cardia | 30 | Yes | 120 | NO | NO | Leiomyoma | 12 |
| 28.M/57 | Cardia-Gastric lesser curvature | 20 | Yes | 115 | NO | NO | GIST( Very low malignant ) | 8 |
| 29.M/59 | Esophago-Cardia | 25 | Yes | 63 | NO | NO | Spindle cell type tumours ( Leiomyoma) | 6 |
| 30.F/45 | Cardia-Gastric lesser curvature | 30 | Yes | 180 | NO | NO | GIST(low malignant) | 8 |
| 31.M/60 | Esophago-Cardia | 30 | Yes | 83 | Yes | NO | Leiomyoma | 6 |
| 32.M/55 | Cardia-Gastric lesser curvature | 30 | Yes | 129 | NO | Nasal hemorrhage | GIST(low malignant) | 6 |
| 33.F/56 | Cardia -Gastric fundus | 25 | Yes | 180 | Yes | SE/ Pt | Leiomyom | 18 |
| 34.F/53 | Cardia -Gastric fundus | 28 | Yes | 161 | NO | SE | Spindle cell type tumours ( Leiomyoma) | 6 |
| 35.M/57 | Esophago-Cardia | 25 | Yes | 63 | NO | NO | Spindle cell type tumours ( Leiomyoma) | 6 |
| 36.F/45 | Cardia-Gastric lesser curvature | 30 | Yes | 180 | NO | NO | GIST(low malignant) | 6 |
| 37.M/57 | Cardia-Gastric lesser curvature | 30 | Yes | 129 | NO | Nasal hemorrhage | GIST(low malignant) | 6 |
| 38.F/58 | Cardia -Gastric fundus | 28/8（two SMTs） | Yes | 161 | NO | SE | Spindle cell type tumours ( Leiomyoma) | 6 |
| 39.M/55 | Esophago-Cardia | 30 | Yes | 83 | Yes | NO | Leiomyoma | 6 |
| 40.M/36 | Cardia -Gastric fundus | 30 | Yes | 185 | NO | NO | GIST(low malignant) | 8 |

^a^ Full muscularis propria was resected for tumors involving the deep muscularis propria；SE subcutaneous emphysema, ME mediastinal emphysema, Pt pneumothorax, Pp pneumoperitoneum.

**Additional file 1: Table S2** Clinicopathological characteristics and treatment outcomes of submucosal tumors of the esophagogastric junction originating from the muscularis propria layer treated by ESE

| **Case**  **Sex/Age.y** | **Tumor**  **Location** | **Tumor**  **Size**  **(mm)average** | **En bloc resection** | **Operation time**  **(min)average** | **Full MP defec**^a^ | **Complication** | **Pathologica diagnosis** | **Follow-up time**  **(month)** |
| --- | --- | --- | --- | --- | --- | --- | --- | --- |
| 1.F/49 | Cardiac-gastric fundus | 10 | Yes | 43 | No | No | Leiomyoma | 24 |
| 2.F/42 | Cardia | 20 | Yes | 50 | Yes | No | Leiomyoma | 24 |
| 3.F/48 | Cardiac-gastric fundus | 10 | Yes | 34 | No | Hemorrhage | Leiomyoma | 24 |
| 4.M/58 | Cardiac-gastric fundus | 15 | Yes | 120 | Yes | Fever | GIST(low malignant) | 18 |
| 5.M/43 | Esophagus-cardia | 25 | No | 150 | No | SE/PT | leiomyoma | 18 |
| 6.F/48 | Cardia | 18 | Yes | 53 | No | No | leiomyoma | 18 |
| 7.M/53 | Cardiac-gastric fundus | 35 | Yes | 125 | Yes | No | GIST  (intermediate malignant) | 18 |
| 8.F/58 | Cardiac-gastric fundus | 12 | Yes | 160 | No | No | GIST(low malignant) | 14 |
| 9.F/66 | Cardiac-gastric fundus | 9 | Yes | 49 | Yes | No | leiomyoma | 14 |
| 10.F/67 | Cardiac-gastric fundus | 10 | Yes | 37 | Yes | No | leiomyoma | 14 |
| 11.F/51 | Cardiac-gastric fundus | 22 | Yes | 65 | No | No | GIST  (intermediate  malignant) | 12 |
| 12.F/56 | Cardiac-gastric fundus | 25 | Yes | 116 | No | No | leiomyoma | 12 |
| 13.M/50 | Cardiac | 12 | Yes | 47 | Yes | No | Schwannoma | 12 |
| 14.F/55 | Cardiac-gastric fundus | 10 | Yes | 31 | No | No | leiomyoma | 10 |
| 15.F/53 | Cardiac-gastric fundus | 8 | Yes | 59 | Yes | No | leiomyoma | 10 |
| 16.M/56 | Cardiac-gastric fundus | 4 | Yes | 67 | Yes | No | leiomyoma | 8 |
| 17.F/76 | Cardiac-gastric fundus | 11 | Yes | 65 | No | No | leiomyoma | 8 |
| 18.F/44 | Esophagus-cardia | 20 | Yes | 69 | No | No | leiomyoma | 8 |
| 19.F/54 | Cardiac-gastric fundus | 10 | Yes | 32 | No | No | leiomyoma | 8 |
| 20.M/27 | Cardiac-gastric fundus | 25 | Yes | 88 | No | No | leiomyoma | 8 |
| 21.F/50 | Cardiac-gastric fundus | 10 | Yes | 29 | Yes | No | leiomyoma | 4 |
| 22.F/52 | Esophagus-cardia | 12 | Yes | 30 | No | No | leiomyoma | 4 |
| 23.F/53 | Cardiac-gastric fundus | 10 | Yes | 39 | No | No | GIST(low malignant) | 3 |
| 24.M/57 | Cardiac-gastric fundus | 10 | Yes | 48 | No | No | GIST(low malignant) | 3 |
| 25.M/57 | Cardiac-gastric fundus | 30 | No | 129 | No | No | GIST  (intermediate malignant) | 3 |
| 26.M/54 | Cardiac-gastric fundus | 35 | Yes | 125 | Yes | No | GIST  (intermediate malignant) | 18 |
| 27.M/52 | Cardiac | 12 | Yes | 47 | Yes | No | Schwannoma | 12 |
| 28.F/57 | Cardiac-gastric fundus | 12 | Yes | 160 | No | No | GIST(low malignant) | 14 |
| 29.F/44 | Cardia | 20 | Yes | 50 | Yes | No | Leiomyoma | 24 |
| 30.F/68 | Cardiac-gastric fundus | 10 | Yes | 37 | Yes | No | leiomyoma | 14 |
| 31.M/58 | Cardiac-gastric fundus | 4 | Yes | 67 | Yes | No | leiomyoma | 8 |
| 32.M/45 | Esophagus-cardia | 25 | No | 150 | No | SE/PT | leiomyoma | 18 |
| 33.F/46 | Cardia | 18 | Yes | 53 | No | No | leiomyoma | 18 |
| 34.F/50 | Cardiac-gastric fundus | 10 | Yes | 34 | No | Hemorrhage | Leiomyoma | 24 |
| 35.F/53 | Cardiac-gastric fundus | 10 | Yes | 31 | No | No | leiomyoma | 10 |
| 36.F/65 | Cardiac-gastric fundus | 9 | Yes | 49 | Yes | No | leiomyoma | 14 |
| 37.F/52 | Cardiac-gastric fundus | 10 | Yes | 29 | Yes | No | leiomyoma | 4 |
| 38.F/50 | Esophagus-cardia | 12 | Yes | 30 | No | No | leiomyoma | 4 |
| 39.F/50 | Cardiac-gastric fundus | 22 | Yes | 65 | No | No | GIST  (intermediate  malignant) | 12 |
| 40.F/57 | Cardiac-gastric fundus | 25 | Yes | 116 | No | No | leiomyoma | 12 |
| 41.M/57 | Cardiac-gastric fundus | 10 | Yes | 48 | No | No | GIST(low malignant) | 3 |
| 42.M/58 | Cardiac-gastric fundus | 30 | No | 129 | No | No | GIST  (intermediate malignant) | 3 |
| 43.F/46 | Esophagus-cardia | 20 | Yes | 69 | No | No | leiomyoma | 8 |
| 44.F/51 | Cardiac-gastric fundus | 10 | Yes | 39 | No | No | GIST(low malignant) | 3 |
| 45.F/47 | Cardiac-gastric fundus | 10 | Yes | 43 | No | No | Leiomyoma | 24 |
| 46.F/74 | Cardiac-gastric fundus | 11 | Yes | 65 | No | No | leiomyoma | 8 |
| 47.F/50 | Cardiac-gastric fundus | 10 | Yes | 32 | No | No | leiomyoma | 8 |
| 48.M/31 | Cardiac-gastric fundus | 25 | Yes | 88 | No | No | leiomyoma | 8 |
| 49.F/55 | Cardiac-gastric fundus | 8 | Yes | 59 | Yes | No | leiomyoma | 10 |
| 50.M/60 | Cardiac-gastric fundus | 15 | Yes | 120 | Yes | Fever | GIST(low malignant) | 18 |

^a^ Full muscularis propria was resected for tumors involving the deep muscularis propria；SE subcutaneous emphysema, ME mediastinal emphysema, Pt pneumothorax, Pp pneumoperitoneum.
